# Boosting BOLD fMRI by K-Space Density Weighted Echo Planar Imaging

**DOI:** 10.1371/journal.pone.0074501

**Published:** 2013-09-10

**Authors:** Mario Zeller, Alexander Müller, Marcel Gutberlet, Thomas Nichols, Dietbert Hahn, Herbert Köstler, Andreas J. Bartsch

**Affiliations:** 1 Department of Radiology, University Clinic, University of Würzburg, Würzburg, Germany; 2 Department of Radiology, Hannover Medical School, Hannover, Germany; 3 Department of Statistics, University of Warwick, Warwick, United Kingdom; 4 Oxford Centre for Functional MRI of the Brain, University of Oxford, John Radcliffe Hospital, Oxford, United Kingdom; 5 Comprehensive Heart Failure Center, University of Würzburg, Würzburg, Bavaria, Germany; 6 Department of Neuroradiology, University of Heidelberg, Heidelberg, Germany; University of Minnesota, United States of America

## Abstract

Functional magnetic resonance imaging (fMRI) has become a powerful and influential method to non-invasively study neuronal brain activity. For this purpose, the blood oxygenation level-dependent (BOLD) effect is most widely used. T_2_* weighted echo planar imaging (EPI) is BOLD sensitive and the prevailing fMRI acquisition technique. Here, we present an alternative to its standard Cartesian recordings, i.e. k-space density weighted EPI, which is expected to increase the signal-to-noise ratio in fMRI data. Based on *in vitro* and *in vivo* pilot measurements, we show that fMRI by k-space density weighted EPI is feasible and that this new acquisition technique in fact boosted spatial and temporal SNR as well as the detection of local fMRI activations. Spatial resolution, spatial response function and echo time were identical for density weighted and conventional Cartesian EPI. The signal-to-noise ratio gain of density weighting can improve activation detection and has the potential to further increase the sensitivity of fMRI investigations.

## Introduction

Echo planar imaging (EPI) is the first choice for blood oxygenation level-dependent (BOLD, [1]) functional magnetic resonance imaging (fMRI) because it provides a T_2_*-sensitive contrast. A whole head volume can be acquired within seconds, while a single slice of the volume is typically acquired in a single echo train after one excitation pulse (single shot EPI). As part of the (pre-)processing after acquisition of the fMRI time-series and prior to its statistical analysis, the data is often smoothed spatially to a variable degree by a Gaussian filter to improve the signal-to-noise ratio (SNR). In hypothesis-driven analyses according to the general linear model (GLM), spatial smoothing prepares the data to better meet basic assumptions of Gaussian random field theory (RFT) for statistical thresholding and inference [2], [3]. Given that anatomical variability across subjects and limits of inter-subject image registration contribute to the variance of fMRI data in common template spaces, spatial smoothing also facilitates studying activations at the group level. According to the matched filter theorem, spatial smoothing improves activation detection if the size of activated clusters and the filter applied for smoothing are well matched.

The highest intrinsic SNR values can be obtained by filtering the k-space proportional to the T_2_* signal decay during the echo train (SNR matched filter, [4], [5]). However, filtering using an SNR matched filter increases the asymmetry of the modulation transfer function (MTF) caused by the signal decay. As a consequence, the spatial response function (SRF) obtained by Fourier transformation of the MTF exhibits strong side lobes, amplifying Gibbs ringing artifacts. This does not occur when a Gaussian filter is used.

One solution to simultaneously increase SNR efficiency while using a Gaussian shaped SRF is to apply acquisition weighting [6], [7]. Here, a Gaussian MTF shape is approximated by sampling the central k-space more often than the periphery. In fMRI experiments, this approach would considerably increase the duration of the EPI readout and is thus not practical. In contrast, k-space density weighting [8] is a technique which allows applying an SNR matched filter while at the same time establishing a desired MTF. The raw data is filtered retrospectively with the SNR matched filter to provide optimal SNR. The resulting MTF/SRF deviations are compensated by acquiring the k-space with a non-Cartesian trajectory. The variable k-space density *ρ(k) = 1/*Δ*k* then acts as an additional parameter influencing the shape of the MTF [9]:
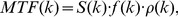
(1)where *S(k)* describes the decaying signal during the echo train and *f(k)* is the retrospectively applied SNR matched filter. The target MTF can in principle be of any form, even be identical to the signal envelope given by the signal relaxation [4]. The effects of the SNR matched filter (*S(k) = f(k)*) on the MTF shape can be compensated by choosing the k-space density according to:



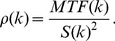
(2)In EPI the variable k-space density is realized by adjusting the phase blip gradient amplitudes proportional to Δ*k = 1/ρ(k)*. Such non-Cartesian k-space density weighted imaging takes no longer to record than a Cartesian acquisition as the number of acquisition steps remains identical and only their position in k-space is varied.

**Figure 1:**
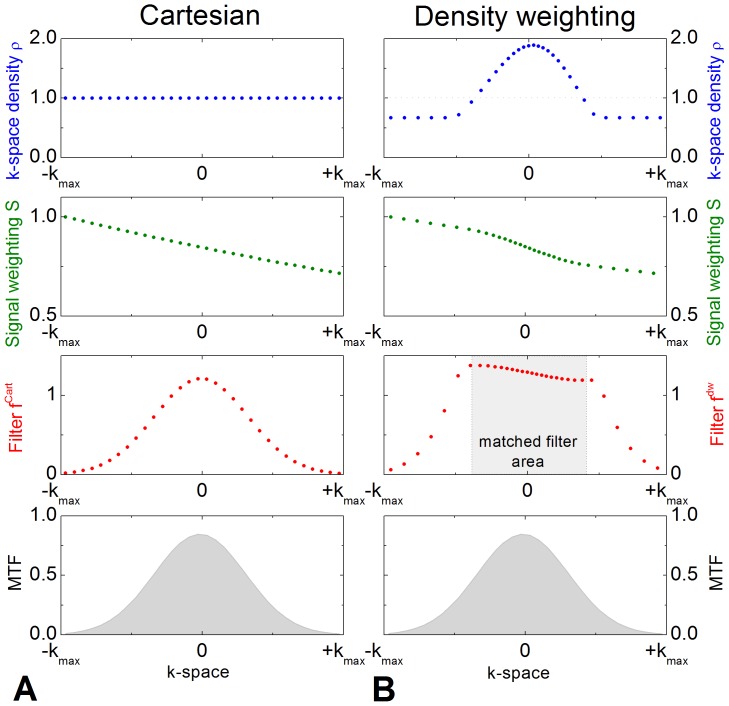
Cartesian (A) and density weighted acquisition (B) for a typical fMRI experiment. The MTF (bottom, grey) results from a multiplication of the k-space density *ρ(k)* (blue, top) with the signal *S(k)* (green) and the filter *f(k)* (red).

Figure 1 shows the formation of the MTF for Cartesian and density weighted EPI. The MTF results from multiplying the signal weighting *S(k)* with the filter *f(k)* and the k-space density *ρ(k)* for the Cartesian (left) and density weighted acquisition (right), respectively. In Cartesian imaging, the k-space density is constant throughout the whole acquisition (blue, top). For fMRI, the signal decays exponentially with T_2_* (green, T_2_* = 50 ms in this example) and is typically multiplied with a Gaussian filter (red). This results in a slightly asymmetric MTF (bottom, grey). For density weighting, this MTF shape can be reproduced by a non-uniform k-space density (blue, top). The signal (green) can be multiplied with an SNR matched filter (red) proportional to the signal decay. In the example shown, the k-space density is limited by a lower bound and thus, the filter deviates from the matched filter case in the k-space periphery. Due to the density variation, the k-space center is oversampled, while the periphery exhibits a k-space density that violates the Nyquist criterion. This violation normally results in incoherent undersampling artifacts. However, those can be avoided by reconstructing the data using parallel imaging for effective density weighted (PLANED) imaging [10], which is essentially a non-Cartesian GRAPPA/PARS algorithm. Due to the non-Cartesian distribution of the k-space density it may occur that the central echo line is shifted for linear acquisitions as used in this study which then results in a changed effective echo time TE_eff_ as compared to a Cartesian acquisition.

Density weighting has already been applied to a variety of MR sequences [4], [8]–[10]. In this work, the technique of k-space density weighting is transferred to EPI. The implications of using the technique with EPI are discussed and the feasibility of its application is demonstrated in phantom and *in vivo* acquisitions with an MTF typically used in fMRI experiments. Finally, potential benefits of the technique are demonstrated by initial fMRI data.

## Methods

### Ethics Statement

The study was approved by the local ethics committee (Ethics Committee at the Faculty of Medicine of the University of Würzburg, reference no 22/11). Written informed consent was obtained from each participant prior to *in vivo* measurements.

### Image Acquisition

Phantom and *in vivo* measurements were performed on a 3 Tesla scanner (Magnetom Trio, Siemens Healthcare, Erlangen, Germany) equipped with a 12-channel head coil. Cartesian and k-space density weighted EPI images were acquired using a single-shot EPI sequence (64×64 matrix, FOV 220×220 mm^2^, slice thickness 3.0 mm, 40 axial slices, TE = 30 ms, TR = 2.2 s, echo spacing 540 µs) with two-fold GRAPPA acceleration (r = 2). A single separate low-resolution calibration data scan for later parallel imaging reconstruction for both methods was recorded at the beginning of each experiment (acquisition time T_acq_ = 2.2 s).

Five healthy volunteers (4 female, 1 male, 4 right-handed, 1 left-handed, age ranging from 24 to 40, mean age 33±7) took part in an fMRI experiment, consisting of a left-hand finger-tapping task. The task was performed in five on/off block cycles starting with rest. To avoid any potential bias of acquiring two different time-courses in separate experiments, Cartesian and density weighted volumes were recorded in an alternating and interleaved fashion. In total, 150 volumes were acquired in 5 min 30 s, resulting in 75 Cartesian and 75 k-space density weighted volumes. The first two volumes were omitted from the analysis to assure steady-state of global magnetization. For temporal SNR comparison (see below), one additional subject (right-handed female aged 40 years) was scanned using the same density weighted/Cartesian EPI acquisition at rest, i.e. without the experimental finger-tapping paradigm.

**Figure 2:**
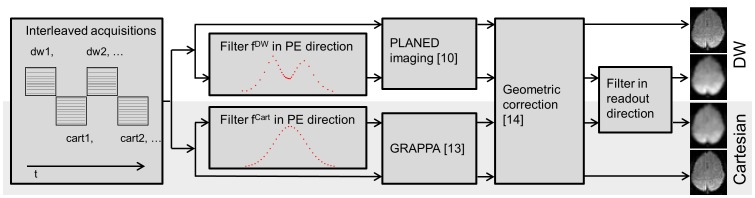
Flow chart of the steps involved in the reconstruction of the fMRI timeseries. The interleaved Cartesian and density weighted datasets are first splitted. Subsequently, unfiltered and filtered images are reconstructed for both acquisition methods, respectively. The filtered images are utilized for statistical processing, whereas the unfiltered images are not SNR efficient and solely created for more accurate motion correction and registration of the filtered data to the anatomical images.

The density weighted sampling was chosen to yield an identical MTF as the Cartesian acquisition after filtering at T_2_^*^ = 50 ms (Figure 1). Thus, SRF and spatial resolution of k-space density weighted and Cartesian EPI were identical. To avoid noise enhancement in the parallel imaging reconstruction, the maximum k-space distance was limited to an additional factor of 1.5 for the density weighted trajectory, which yielded a maximum k-space undersampling of 3 in combination with the two-fold GRAPPA acceleration in the k-space periphery. A further constraint for the trajectory was to obtain identical echo times for density weighted and Cartesian acquisitions. This alters the shape of the matched filter (see Figure 2) and thus provides not the best achievable solution for k-space density weighted images but was nevertheless introduced in order to keep density weighted and Cartesian acquisitions fully comparable.

To allow for off-resonance correction of the fMRI data, a low-resolution multi-echo reference scan was also acquired prior to the fMRI experiment [11]. The scan comprised 32 phase encoding steps without GRAPPA acceleration and thus 32 repetitions (acquisition time T_acq_ = 70 s) and had otherwise identical parameters as the fMRI scan.

A three-dimensional magnetization prepared rapid acquisition gradient echo (MPRAGE, 0.9 mm^3^ isotropic resolution, 208 slices, TE = 2.24 ms, TR = 1720 ms, TI = 900 ms, 9° flip angle) scan was recorded as a high-resolution anatomical image for accurate image registration across subjects to MNI152-template space.

### Image Reconstruction

Image reconstruction was performed using MATLAB (The Mathworks, Natick, MA, USA). Figure 2 shows the steps involved in the reconstruction of Cartesian and density weighted data, respectively.

The density weighted k-space data was first multiplied with the SNR matched filter described earlier in the text. Subsequently a fully sampled k-space was obtained using the non-Cartesian PLANED imaging algorithm [10]. In our implementation, two evenly spaced intermediate k-space positions were calculated between two acquired k-space lines. Those positions were then brought onto a Cartesian grid together with the acquired k-space lines using convolution gridding without density compensation [12].

The Cartesian k-space data was first multiplied with a Gaussian filter (with σ = 0.85 px in image space). A fully sampled k-space was then reconstructed by standard Cartesian GRAPPA [13].

Both reconstructed data sets were then off-resonance corrected utilizing a conjugate phase multifrequency interpolation method [14]. The required field map was derived from the previously acquired multi-echo reference scan [11]. Finally, the Gaussian filter was also applied in read direction of the two datasets and the separate coil images were combined by taking a pixel-by-pixel coil-sensitivity weighted sum [15].

In order to improve motion correction and image registration during the fMRI analysis (described in the following section), the unfiltered time-series were also obtained for both methods by performing the steps described above without applying any filters during the reconstruction.

### Data Preprocessing and Statistical Analysis

K-space density weighted and Cartesian EPI were further processed and statistically analysed using FSL 5.0 (http://fsl.fmrib.ox.ac.uk/fsl/fslwiki/) [16], [17]. Unfiltered time-series were corrected for motion using mcflirt (part of FSL; [18]). The rigid-body (6 degrees of freedom) inter-volume registration matrices obtained for correction were then applied to the volumes of the filtered time-series. Subsequently, brain extraction (using BET, also part of FSL; [19]) and high-pass temporal filtering (cutoff at 70 s, slightly above the block design’s on-off cycle time) were performed. Time-series statistical analysis was carried out according to the General Linear Model (GLM) within FEAT using FILM prewhitening (both part of FSL) with local autocorrelation correction [20]. The time-series model was set up by convolving the block design of the finger tapping (see above) with FSL’s canonical hemodynamic response function (HRF) using the default gamma function and including a temporal derivative (in FSL’s GLM GUI). Because the mean 3D signal differs between density weighted and Cartesian volumes, the joint model for their interleaved recording was then separated for the two types of EPI acquisitions (by extracting the volume-wise entries from the relevant design.mat files and re-entering these as explanatory variables into FEAT without re-convolution). Thereby, grand-mean 4D intensity scaling by a single multiplicative factor was performed separately for the density weighted and Cartesian EPI time-series at the first level while 3D intensity normalization to a preset constant was avoided to ensure that the analyses are valid at the second level. Upon boundary-based within-subject registration of the functional and anatomical scans [21] and subsequent non-linear registration of the structural scans to the MNI152 template using FNIRT (part of FSL; [22]), group-level analysis across the n = 5 subjects was carried out by a Fixed-Effects (FE) model at the higher (i.e. second) level using FEAT [23]. FE error variances are the variances from the first level, and weighting is introduced into a standard weighted FE model by allowing these to be heteroscedastic. Statistical inference from FE modeling is very sensitive to detect activations at the higher level, yet the reported results are with respect to the sample of subjects studied and are not generalized to the wider population from which these are drawn. Z- (i.e. Gaussianised T-) statistic images were thresholded using clusters determined by Z>2.3 and a family-wise error rate (FWER)-corrected cluster significance threshold of p≤0.05 [24], both for exemplary first-level data as well as the second level FE analysis. For the latter, pre-threshold masking was performed in order to limit the number of multiple comparisons. The binary pre-threshold mask included pre- and postcentral gyrus and supplementary motor area (SMA) as derived from the Havard-Oxford Cortical Structural Atlas in MNI152 space (part of FSL; each of the three structures thresholded at 25% probability). Group-level mean FE fMRI results for k-space density weighted and Cartesian EPI were separately projected to and visualized on the MNI152 pial surface using mri_vol2surf and tksurfer, both part of FreeSurfer 5.2.0 (http://surfer.nmr.mgh.harvard.edu/fswiki; [25], [26]). The differential effect of density weighted vs. Cartesian EPI is rendered in MNI152 volume space. All sectional brain images are displayed in radiological convention, with the left side of the brain shown on the right side in the figures.

### Voxel-wise Quantification of SNR and Relative BOLD Signal Change

Spatial response functions were obtained by deriving the edge spread functions of phantom acquisitions. The latter were taken from a row perpendicular to a sharp edge and in an area of constant signal intensity.

Spatial SNR was determined by a pseudo multiple replica method [27] utilizing noise scans acquired at the beginning of each examination, and simulations of spatial SNR for different T_2_* were performed for Cartesian and density weighted acquisitions as described in [9]. Spatial SNR was then empirically estimated for all voxels of native Cartesian and density weighted EPI (from the 4^th^ and 5^th^ volume of the time-series, corrected only for motion and geometric distortions *without* further data preprocessing) in 10 consecutive slices centered around the handknob. Similarly, temporal SNR was estimated voxel-wise from the same 10 motion- and distortion-corrected slices by the ratio of the mean signal to the standard deviation over time. In order not to bias temporal SNR by task-related functional activation, the union of significantly activated voxels from the first-level analyses of Cartesian and density weighted EPI were excluded. This is compared to temporal SNR in the resting-state data of the additional subject where all voxels were retained.

Signal change values were also quantified on a voxel-to-voxel basis by BOLD response amplitudes, averaged of the duty cycle of the task, within a spherical region-of-interest (ROI) of 25 mm diameter centered to the right precentral handknob [28], i.e. contralateral to the finger tapping (using fslmaths, fslstats and featquery, all part of FSL; and MATLAB). While this ensures a priori that fMRI activation is indeed present within the ROI, it avoids any second-level bias and circularity [29] in extracting the BOLD response magnitudes. Given that the anatomically predefined handknob ROI was relatively large and therefore contained non-activated voxels (for which no effective difference between Cartesian and density weighted EPI can be expected or achieved), the lower 10^th^ percentile of BOLD signal change values was excluded for both EPI variants and the remainder of the values was expressed relative to the mean of conventional Cartesian EPI as relative signal change (rSC). Additionally, time-courses of percentual signal change in density weighted and Cartesian EPI were extracted from the differential FE cluster, as backprojected to native EPI space and averaged across subjects (using fslmeants and featquery; part of FSL). Although this was based on unfiltered raw data prior to any preprocessing, the corresponding effect is informed by the second level and only displayed here for illustration, i.e. it should not be used as the basis for future power analyses.

Spatial SNR, temporal SNR and rSC of density weighted vs. Cartesian EPI were then compared to each other on a voxel-wise basis using Bland-Altman plots [30]. Based on these data we computed a one-sample t-test on the mean difference in density weighted vs. Cartesian data per subject. Due to the small sample size (n = 5 subjects) we used a non-parametric permutation test which does not depend on any normality assumptions. It consists of repeatedly re-computing the t-test after randomly flipping the sign of mean difference of each subject [31]. Since there are 2^5^ = 32 ways to flip the signs on the 5 subject’s differences, the smallest possible permutation p-value that can be achieved is 1/32 (p = 0.03125).

Furthermore, first-order autocorrelation maps were generated and temporal AR(1) coefficients were extracted (using fslmaths and fslstats; both part of FSL). The number of resolution elements (RESELs) according to Gaussian RFT was obtained in dividing the volume by the RESEL size entry (both stored in FEAT’s smoothness file), and the cubic root of the RESEL size was taken as the geometric mean of the underlying smoothness. Data and implemented methods are available upon request.

## Results

**Figure 3:**
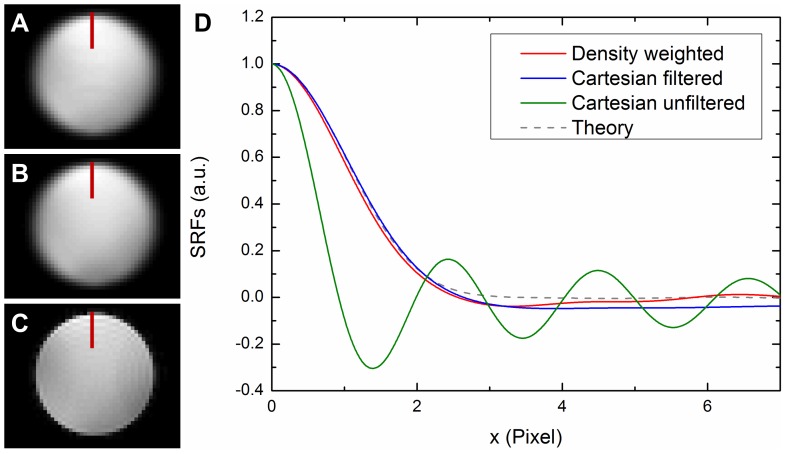
Phantom images and corresponding spatial response functions. Images were reconstructed from k-space density weighted (A), Cartesian (B) and unfiltered Cartesian acquisition (C). Spatial response functions (D) were obtained from the edge spread functions indicated by the red bars.

Figure 3 shows images of a phantom acquired utilizing density weighted (A) and Cartesian imaging with retrospective filtering (B) as well as Cartesian imaging without any filtering (C). The unfiltered image exhibits clearly visible Gibbs ringing artifacts. Spatial response functions obtained from those phantom images by deriving the edge spread functions (indicated by red bars) are shown in (D). The SRF of the unfiltered Cartesian acquisition (green) exhibits extensive side lobes. Those are eliminated in the density weighted (red) and Cartesian filtered SRFs (blue) which are nearly identical and correspond well with the theoretically expected SRF (dashed gray line). The FWHM is broadened by a factor of 2 compared to the unfiltered SRF, which corresponds well to the FWHM of the Gaussian filter with σ = 0.85 px in image space. Here, the spatial SNR advantage of density weighted vs. Cartesian EPI amounted to 13% which corresponds very well to the theoretical prediction of 14% [4], [9].

**Figure 4:**
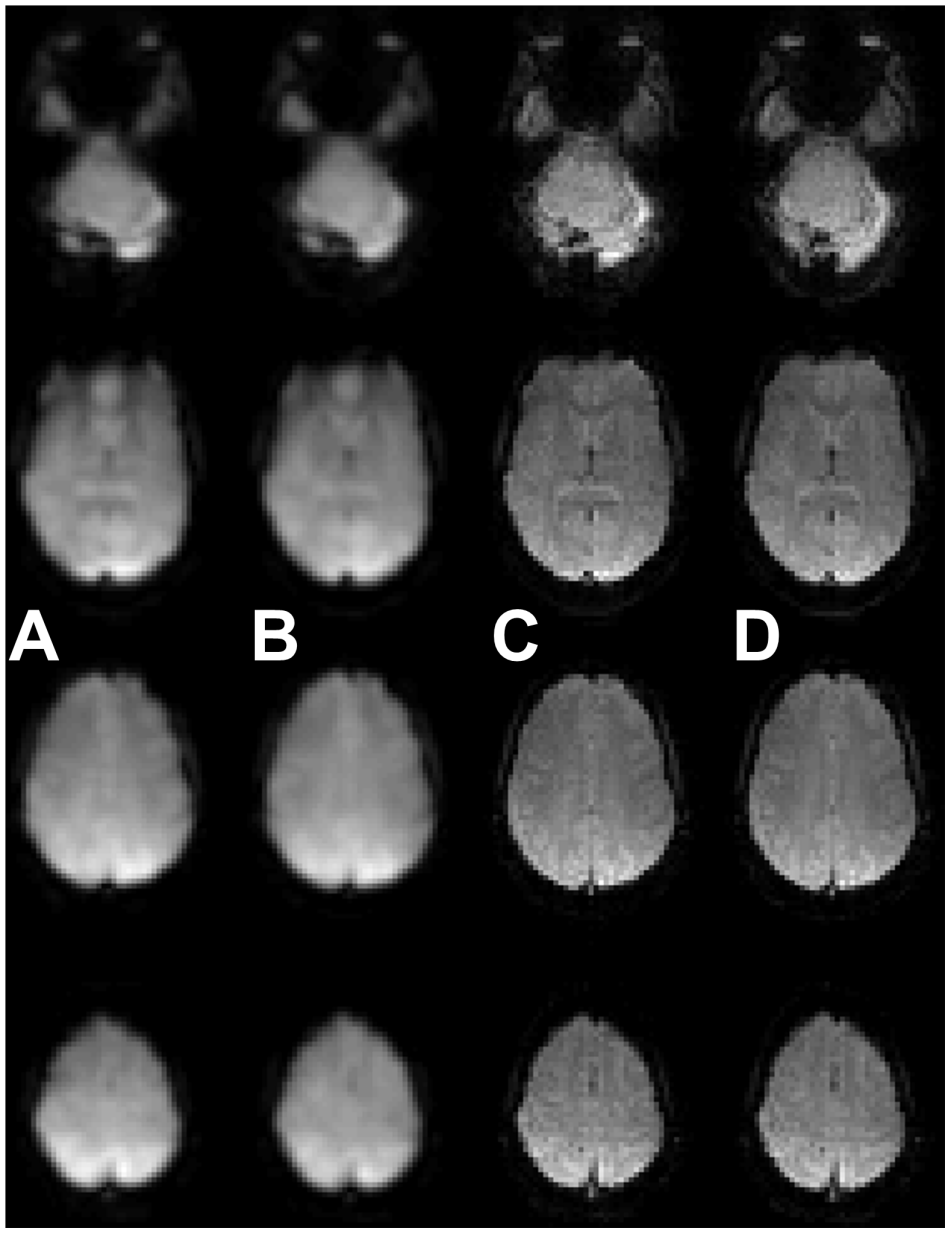
Representative slices of the brain of a healthy volunteer. Images are shown for k-space density weighted (A), Cartesian filtered (B), unfiltered density weighted (C) and unfiltered Cartesian reconstructions (D). Cartesian and density weighted images correspond well in geometry and contrast.

Figure 4 shows selected slices from the *in vivo* measurement of a representative healthy volunteer. Density weighted (A) and Cartesian filtered acquisitions (B) were reconstructed as described in Figure 2. Density weighted (C) and Cartesian EPI reconstructions (D) without filtering for later motion correction and image registration are also shown to demonstrate the comparable geometric shape of both acquisition methods. Both were corrected for geometric distortions based on a multi-echo reference scan (cf. Figure 2). Geometric distortions in the phase-encoding direction (here: anterior-to-posterior) arise from local magnetic field inhomogeneities caused by magnetic susceptibility gradients in neighboring tissues, especially at the skull base.

**Figure 5:**
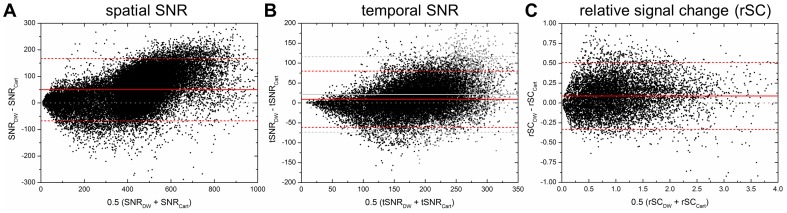
Bland-Altman difference plots of quantitative parameter gains. Gains are plotted on a voxel-to-voxel basis for spatial SNR, temporal SNR and relative signal change (rSC; scaled to the mean Cartesian value) for density weighted (DW) over Cartesian (Cart) EPI in n = 5 subjects. Red solid lines represent the mean difference across voxels and subjects, red dashed lines ±1.96 × the standard deviation (SD; 95% limits of agreement for each comparison). The dotted gray line represents identity (no difference). Average increases in spatial SNR (12.4%, t = 15.68), temporal SNR (5.5%, t = 2.46) and rSC (8.6%, t = 2.63) were consistent and statistically significant (p<0.03125; based on mean within-subject differences). Gray data points and corresponding gray lines in the tSNR plot represent values of one additional subject measured at rest for comparison.

Bland-Altman plots of voxel-wise spatial and temporal SNR as well as relative BOLD signal change (rSC) values of density weighted and Cartesian acquisitions are shown in Figure 5 for all subjects. The plotted mean difference values were consistently above zero for all data shown: Mean gain in spatial SNR amounted to 12.4% (standard error 6.6%, t = 15.68), mean gain in temporal SNR amounted to 5.5% (standard error 9.6%, t = 2.46) and mean gain in relative signal change (rSC) amounted to 8.6% (standard error 8.8%, t = 2.63). Each of these gains in spatial SNR, temporal SNR and rSC was statistically significant (p = 0.03125), i.e. density weighting enhanced average spatial and temporal SNR as well as fMRI signal change over conventional Cartesian EPI. The gain in temporal SNR of the resting-state dataset acquired from the additional subject amounted to 10.6%, i.e. almost twice as high as in the task fMRI data. First-order autocorrelations of the time-series were very similar for Cartesian and density weighted acquisitions and are not presented separately here. At the second level, density weighted and Cartesian data were of similar smoothness (4.0 vs. 4.1 mm FWHM) and number of RESELs (3637 vs. 3451 RESELs in MNI152 standard space).

**Figure 6:**
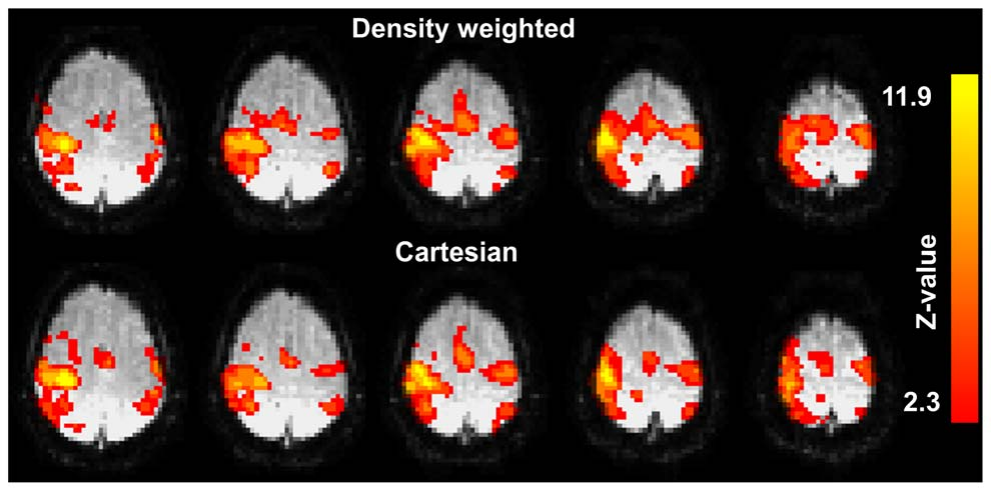
First-level fMRI results. Five consecutive slices of the statistical activation images thresholded using clusters (determined by Z>2.3 and a FWER-corrected p≤0.05) of the subject presented in Fig. 4 for the density weighted and the Cartesian acquisition.

**Figure 7:**
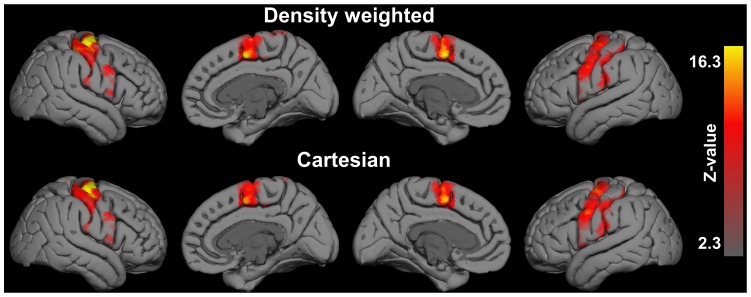
Second-level fixed-effects (FE) fMRI results - Mean activation. Evoked by left-hand finger tapping in n = 5 subjects as detected by density weighted (top) and Cartesian (bottom) EPI acquisitions (all thresholded using clusters determined by Z>2.3 at a FWER-corrected p≤0.05 and projected to the pial surface of the MNI152 template).

Figure 6 shows five consecutive slices of the statistical activation maps, thresholded using clusters determined by an initial cluster forming threshold of Z>2.3 and a final FWER-corrected cluster significance threshold of p≤0.05, from the first level GLM analysis of the same representative subject shown in Figure 4 performing the left-hand finger tapping experiment using density weighted and Cartesian EPI recordings. Figure 7 displays the mean FE activations, again thresholded using clusters determined by Z>2.3 and a FWER-corrected cluster significance threshold of p≤0.05, as detected by density weighted and Cartesian EPI acquisitions at the second level in our sample of n = 5 subjects. Both of these figures demonstrate the feasibility of fMRI by k-space density weighted EPI and that its results closely match those obtained by conventional Cartesian EPI acquisitions. At the first level, density weighting detected more extensive sensorimotor and SMA activations than Cartesian EPI (Fig. 6). At the second level, higher activation levels became apparent around the postcentral gyrus, i.e. the primary sensory cortex, contralateral to the tapping fingers at the level of the handknob and in the SMA (Fig. 7). Peak activations were located around the handknob area contralateral to the tapping hand in both instances (MNI152 coordinates × = 40, y = −22 and z = 54 mm for density weighting and z = 50 mm for Cartesian EPI) and higher for the density weighted compared to the Cartesian acquisitions (max. FWER-corrected -log10(p) = 33.9 vs. 23.5 for the clusters at these very coordinates), with density weighting detecting 106 more activated voxels with a total volume of 848 mm^3^ in MNI152 space. Table 1 lists four corresponding local FE activation maxima from density weighted and Cartesian EPI acquisitions for the group of n = 5 subjects performing the left-hand finger tapping task. Statistical t-values at these local maxima were consistently higher for density weighted as compared to Cartesian EPI while being no more than two voxel coordinates apart.

**Table 1:** Corresponding local activation maxima from second level fixed-effects (FE) analyses of the mean activation evoked by left-hand finger tapping as detected by density weighted and Cartesian EPI in n = 5 subjects.

Density weighted				Cartesian				
t-stats	x	y	z	t-stats	x	y	z	Anatomical Label*
30.4	40	−22	54	27.9	40	−22	50	R Post−/Precentral G.
27.1	42	−20	64	25.6	42	−20	66	R Pre−/Postcentral G.
23.2	32	−12	68	22.4	32	−10	70	R Precentral G.
20.1	−2	−2	52	17.9	−2	−2	54	SMA

R … right, SMA … supplementary motor area, G. … gyrus.

x, y, z …MNI152 coordinates [mm].

t-stats … statistical t-values.

* based on the Havard-Oxford Cortical Structural Atlas (part of FSL).

**Figure 8:**
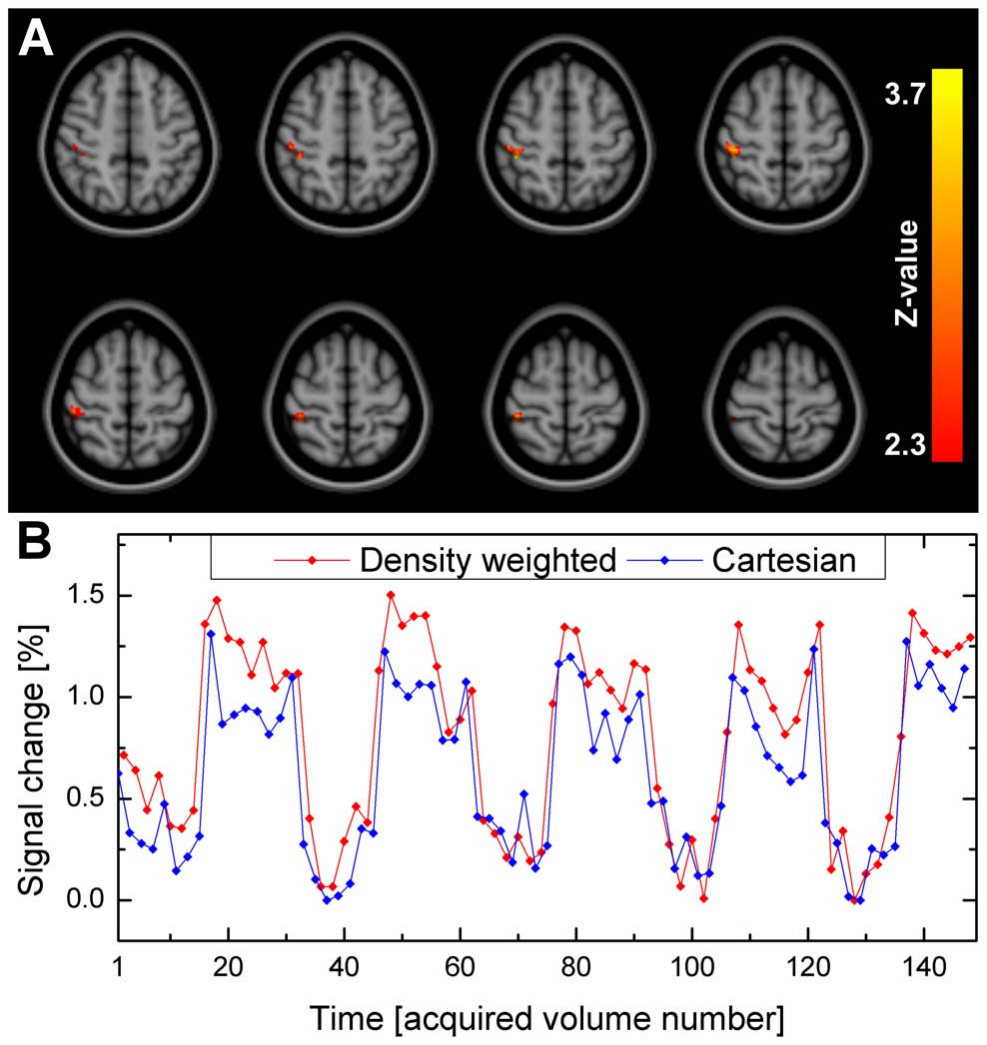
Second-level fixed-effects (FE) fMRI results - Differential contrast. (A) Revealing a cluster of significantly increased activation detected by k-space density weighted compared to conventional Cartesian EPI for left-hand finger tapping in n = 5 subjects (thresholded using clusters determined by Z>2.3 at a FWER-corrected p≤0.05 and displayed in MNI152 standard space). In the opposite, no areas of increased activation detected by Cartesian over density weighted EPI were found. (B) Time-courses within this cluster (extracted from raw data prior to further processing and averaged across n = 5 subjects) reveal increased percentual BOLD signal changes of density weighted compared to Cartesian EPI for each of the five blocks of the finger-tapping task.

Figure 8A shows the differential effect of higher activations detected by density weighted as compared to Cartesian EPI according to the FE analysis across all n = 5 subjects examined. Here, significantly higher activations were revealed in a cluster of 117 voxels comprising 936 mm^3^ in standard space, centered to the right postcentral gyrus at the level of the handknob (MNI152 coordinates × = 42, y = −32, z = 56 mm, FWER-corrected -log10(p) = 0.018, Z-max = 3.76). Figure 8B depicts the associated time-courses of density weighted and Cartesian acquisitions, averaged over this cluster in the unfiltered raw EPI data of the n = 5 subjects. This illustrates the increased percentual BOLD signal changes of density weighted compared to Cartesian EPI that were detected during each of the five blocks of the finger tapping task. Conversely, no areas of significantly increased fMRI activation during conventional Cartesian as opposed to density weighted EPI were found.

## Discussion and Conclusion

Density weighting was successfully implemented for EPI and evaluated in phantom and fMRI experiments. Even with the constraints set for the density weighted k-space trajectory (i.e. a limitation of the additional k-space undersampling factor to 1.5 and identical echo time as the Cartesian acquisition) a considerable spatial and temporal SNR improvement over conventional Cartesian imaging can be realized while maintaining identical spatial resolution. Data from a simple finger tapping experiment suggest that density weighting may actually enhance the detection of fMRI activations.

### Voxel-wise Quantification of SNR and Relative BOLD Signal Change

As demonstrated in phantom acquisitions, the SRFs and thus the spatial resolution are identical for density weighted and Cartesian acquisition with retrospective Gaussian filtering. The SNR advantage of 13% measured in a homogeneous phantom *in vitro* and the SNR advantage of 12.4% measured *in-vivo* (cf. Figure 6) correspond well with the theoretical prediction of 14% [4], [9]. Deviations from the theoretical value may arise from imperfect voxel-to-voxel correspondence between Cartesian and density weighted reconstructions, noise enhancement by parallel imaging [27] or inhomogeneity-induced k-space shifts [32]. Slight off-center shifts of the k-space maximum can result from residual B_0_ inhomogeneities and were observed in some subjects [32]. Due to the different shape of the filters applied to Cartesian and density weighted data (cf. Figure 2), the Cartesian or the density weighted data may be more sensitive to inhomogeneity-induced shifts depending on the shift direction, respectively. This will potentially result in a decreased or increased SNR gain of density weighted over Cartesian EPI.

For turbo spin echo sequences it has been shown that the actual SNR gain of density weighting vs. Cartesian imaging depends on the tissue relaxation parameters [9]. It increases if the relaxation time is shorter than assumed for the calculation of the density weighted k-space sampling and decreases if the relaxation time is longer. However, with the sequence parameters used in this study, the influence of tissue T_2_* deviations from 50 ms assumed for the calculation will be very small. For example, the expected SNR gain is still 13.8% for T_2_* = 500 ms (vs. 14.0% for T_2_* = 50 ms). This minimal variation can be explained by the relatively small signal decay between the first and the last echo in the echo train (by 28.5% for T_2_* = 50 ms). Thus, the signal shape has only a modest impact on the calculation of the density weighted k-space sampling. The SNR advantage increases significantly only for very short T_2_* (<15 ms) with the parameters used in this study. However, as in turbo spin echo imaging the SNR variation can be significant for differently chosen sequence parameters (for instance longer echo trains or if the confound of fixed echo time is omitted).Also, it is known that BOLD signal amplitude varies for intra- and extravascular contributions [33]–[35] depending on the echo time TE. Even though the echo time was kept identical for Cartesian and density weighted acquisition, the different k-space sampling patterns possibly still influence the amplitude of the BOLD response to a small amount depending on the size of the structure of interest.

The gain in temporal SNR was lower than the gain in spatial SNR (cf. Figure 5). This corresponds to earlier observations and may be assigned, on the one hand, to the influence of physiological noise [36], [37]. As shown by Triantafyllou et al. [37], the gain in temporal SNR can be increased by adjusting the acquisition parameters. For density weighted acquisitions, this will be subject of further investigations. On the other hand, the gain in temporal SNR amounted to 10.6% in the resting-state measurement of the additional subject and was thus closer to the spatial SNR value than the temporal SNR values obtained by excluding the areas of activation in our finger-tapping task fMRI (5.5%). Here, it has to be taken into account that fMRI data recorded at rest do not just contain physiological noise but also significant fluctuations of neuronal activity, i.e. of the so-called resting-state networks (RSNs) [38], [39] such as the default-mode network (DMN) which has been shown to be deactivated by attention-demanding tasks [40]. RSN activations can be expected to increase temporal SNR in baseline scans while RSN deactivations may decrease temporal SNR in fMRI data with task-related activations excluded. This would correspond to our observations.

Within the predefined ROI centered to the right precentral handknob, an average gain in fMRI response magnitudes of 8.6% was achieved for all voxels above the lower 10^th^ percentile of BOLD signal change values from the density weighted and Cartesian EPI data. This value can be considered a conservative estimate of what is attainable by k-space density weighted EPI for BOLD fMRI. Mean activation of local maxima (cf. Table 1) was enhanced by up to 12% which matches the theoretical prediction more closely but does not yet cover the area where the strongest enhancement over conventional EPI was observed (Fig. 8A/B).

The relationship between the detectability of fMRI activations and temporal SNR is highly non-linear [41], and increases in spatial SNR themselves improve temporal SNR less than proportionally [37]. The presence of correlated, non-stationary noise of RSNs and other physiological sources implies that temporal SNR does not simply increase with the square root of the number of time-points recorded. Considering that the number of time-points necessary to detect BOLD signal changes of a given effect strength non-linearly decreases the higher the temporal SNR [41], k-space density weighted EPI may be useful to shorten the scan time required for fMRI experiments. This would be particularly beneficial for clinical applications where patient performance and compliance are often limited [42] but clearly needs to be supported by separate experimental data. Furthermore, density weighting may be expedient for fMRI at higher image resolutions. Higher spatial resolutions reduce physiological-to-thermal noise ratios [37] where smoothing improves temporal SNR without augmenting physiological noise [43].

At this point, it must be emphasized that the theoretically predicted and *in vitro* confirmed SNR gain of density weighted EPI did not uniformly translate into improved detection of global fMRI activations *in vivo*. Obviously, not all areas that activated on average in conventional Cartesian recordings (cf. Fig. 7) revealed an enhanced activation level upon k-space density weighting. Instead, significantly increased activation of density weighted EPI was detected in a limited cluster of the postcentral gyrus located behind the top four local mean activation maxima (cf. Fig. 8, Table 1). This may be due to a variety of reasons. First of all, spatial and temporal SNR are likely to vary across space, for example due to inhomogeneity-induced k-space shifts [32], physiological noise [36] or noise enhancement by parallel imaging [27]. Second, it will be hard and require much larger samples to demonstrate an advantage of density weighting in areas of high activations which exhibit a strong BOLD response *per se*. Notably, the cluster we detected in favor of density weighted acquisitions does not project on the precentral motor handknob of highest mean activation (Fig. 7) but the postcentral cortex which gets also, yet less activated by primary sensory stimulation during contralateral finger tapping (Fig. 8). In this context, it has to be stressed that no areas of significantly increased activation were detected by conventional Cartesian compared with density weighted EPI.

These issues require further study, larger samples and mixed-effects analyses to be substantiated and generalized to the population level. However, our data demonstrate the general feasibility of fMRI by k-space density weighted EPI and indicate, as a proof of principle, its potential benefits of boosting SNR and the sensitivity of activation detection.

### Implementation

In the presence of B_0_ inhomogeneities Cartesian EPI acquisitions typically exhibit geometric distortions caused by a phase accrual during the echo train [44]. Distortions arising in density weighted EPI acquisitions additionally involve changes in the shape of the SRF [45]. These artifacts are caused by the non-linear dependence of the k-space position on the sampling time introduced by the non-Cartesian k-space sampling.

These distortions can be corrected utilizing conjugate phase based methods. In this work, a multi-frequency interpolation method [14] based on a multi-echo reference scan [11] was used for correction of Cartesian and density weighted reconstructions. However, the inhomogeneity effects were rather small for Cartesian and density weighted reconstructions because of the short echo train used in this work.

In fMRI, the echo time TE_eff_ of the EPI acquisition influences the activations that are detectable [36], [46], [47]. As already demonstrated, density weighting has revealed higher activation levels even at shorter echo times than the Cartesian reference acquisition [48]. In that study, the spatial resolution was identical but the shape of the SRF of the density weighted and Cartesian acquisitions was not the same. In the work presented here, both SRF and echo time were - in addition to spatial resolution - kept identical for density weighted and Cartesian acquisition. Thereby, possible effects of different SRFs or echo times on the results were excluded as confounds while at the same time a set of relatively realistic acquisition parameters for single-subject and group-fMRI studies is provided.

Setting the density weighted k-space sampling under the constraint to yield an identical echo time as the Cartesian acquisition allows for a fair comparison between the two methods. However, the constraint results in a reduced SNR advantage (14.0%) compared to the unconstrained case (17.6%). This deviation from the ideal SNR matched filter and thus the reduction in maximally achievable SNR advantage will be considerably higher for longer echo trains.

It could be advantageous to leave the echo time unconstrained for high resolution imaging or imaging of tissue compartments with short T_2_^*^ or large susceptibility gradients (such as the inferior frontal lobe adjacent to the frontal paranasal sinuses) in order to allow for shorter echo times and thus higher achievable SNR as well as reduced intra-voxel dephasing. Another possible application is fMRI at higher field strengths with shorter T_2_^*^ and high spatial image resolutions (see above). Future studies will have to evaluate whether the increased SNR of k-space density weighted EPI is able to compensate for decreasing T_2_* contrast in fMRI with shorter than usual TEs.

GRAPPA reconstruction calibrations for both Cartesian and k-space density weighted acquisitions were performed utilizing a separately acquired low-resolution EPI scan. As demonstrated earlier [9], the calibration for density weighting can also be performed using the oversampled k-space part in an auto-calibrating manner. In contrast to Cartesian parallel imaging, a separate calibration scan would be redundant. Additionally, a re-calibration could be performed anytime throughout the whole acquisition to account for intervening effects such as subject motion.

In the implementation presented here, geometric distortion correction was performed on the data already filtered in phase encoding direction and motion correction parameters were derived from unfiltered reference images and applied on the filtered data. This was mainly due to the different software involved in reconstruction (MATLAB) und statistical analysis (FSL). In typical fMRI pre-processing scenarios, the filtering is performed as a final preprocessing step and we acknowledge that the order of our image reconstruction and preprocessing steps can be further optimized. For example, an iterative approach would be conceivable to also account for movement-by-susceptibility interactions while correcting for geometric distortions and motion [49].

### Other Work

Recently, an implementation similar to density weighting which uses read-out gradients with modulated amplitudes to vary the k-space density has been presented [50]. That implementation is very demanding in terms of the scanner hardware requiring an additional magnetic field monitoring with field probes and subsequent k-space trajectory correction in the post processing. In contrast, the implementation presented here achieves the k-space density variation by simply adjusting the phase blip gradient moments. The implementation into existing sequence codes is very easy and uncritical to the scanner hardware. Thus, no additional field monitoring and correction is necessary, making it straightforward to implement density weighting at other scanner sites. However, an additional SNR gain is expected when density weighting can be applied into two k-space directions simultaneously.

Density weighting does not preclude but can be combined with other acceleration techniques. As demonstrated in this work, density weighting can, for example, be used along with parallel imaging techniques to accelerate the imaging process. As density weighting only changes the phase blip gradient moments, a combination with techniques which improve temporal resolution, such as simultaneous echo refocusing [51], blipped CAIPIRINHA [52] or multiplexed imaging [53] as a combination of both is also possible. Notably, density weighting imposes no restrictions on the MTF shape but can also be used to improve the SNR without altering the original SRF. This principle has already been employed to the SR-FLASH sequence [4] and could also be an option for spin echo EPI and applications like diffusion weighted imaging to improve SNR without compromising spatial resolution.

### Conclusions

K-space density weighting has been applied successfully to echo planar imaging (EPI) and demonstrated higher signal-to-noise ratio (SNR) than Cartesian imaging in phantom and *in vivo* experiments. Even at identical SRF and echo time, it provided significant spatial and temporal SNR advantages over Cartesian acquisitions. In a finger-tapping task of five subjects, a significant boost of local fMRI activations was detected. At identical echo times, k-space density weighting may therefore provide an attractive alternative to standard Cartesian fMRI acquisitions.
